# A Mechanism Exploration for the Yi-Fei-San-Jie Formula against Non-Small-Cell Lung Cancer Based on UPLC-MS/MS, Network Pharmacology, and *In Silico* Verification

**DOI:** 10.1155/2023/3436814

**Published:** 2023-01-09

**Authors:** Leihao Hu, Canfeng He, Aier Mo, Xingkai Zhan, Caizhi Yang, Wei Guo, Lingling Sun, Weiwei Su, Lizhu Lin

**Affiliations:** ^1^School of the First Clinical Medicine, Guangzhou University of Chinese Medicine, Guangzhou, Guangdong, China; ^2^Dongguan Hospital of Integrated Chinese and Western Medicine, Dongguan, Guangdong, China; ^3^School of Basic Medicine, Guangzhou University of Chinese Medicine, Guangzhou, Guangdong, China; ^4^Oncology Center, The First Affiliated Hospital of Guangzhou University of Chinese Medicine, No. 16 Airport Road, Guangzhou, Guangdong 510405, China; ^5^Guangdong Engineering & Technology Research Center for Quality and Efficacy Reevaluation of Post Market Traditional Chinese Medicine, Guangdong Provincial Key Laboratory of Plant Resources, School of Life Sciences, Sun Yat-sen University, No. 135, Xingang Xi Road, Guangzhou, Guangdong 510275, China

## Abstract

Non-small-cell lung cancer (NSCLC) is one of the most prevalent cancers worldwide. A Yi-Fei-San-Jie formula (YFSJF), widely used in NSCLC treatment in south China, has been validated in clinical studies. However, the pharmacological mechanism behind it remains unclear. In this study, 73 compounds were identified using ultraperformance liquid chromatography-tandem mass spectrometry (UPLC-MS/MS), with 58 enrolled in network pharmacology. The protein-protein interaction network, functional enrichment analysis, and compound-target-pathway network were constructed using 74 overlapping targets from 58 drugs and NSCLC. YFSJF has many targets and pathways in the fight against NSCLC. PIK3R1, PIK3CA, and AKT1 were identified as key targets, and the PI3K/AKT pathway was identified as the key pathway. According to the Human Protein Atlas (THPA) database and the Kaplan–Meier Online website, the three key targets had varying expression levels in normal and abnormal tissues and were linked to prognosis. Molecular docking and dynamics simulations verified that hub compounds have a strong affinity with three critical targets. This study revealed multiple compounds, targets, and pathways for YFSJF against NSCLC and suggested that YFSJF might inhibit PIK3R1, PIK3CA, and AKT1 to suppress the PI3K/AKT pathway and play its pharmacological role.

## 1. Introduction

Lung cancer is the most frequent and leading cause of cancer death, with NSCLC accounting for most cases. Fighting against lung cancer is still a major issue. According to the World Cancer Report 2020 published by the International Agency for Research on Cancer (IARC), 2.1 million new lung cancer cases and 1.8 million deaths worldwide were reported in 2018, accounting for 11.6% of cancer cases and 18.4% of cancer deaths [1].

The most common treatments for lung cancer include surgery, chemotherapy, radiotherapy, targeted therapy, and immunotherapy [2]. With the development of clinical studies, a combination of different therapies has been a trend for present and future treatment, especially for advanced lung cancer patients [3–8]. Many hospitals in China use a combination of contemporary pharmacological medications and traditional Chinese medicine (TCM). The Yi-Fei-San-Jie formula (YFSJF), formerly known as the Yi-Qi-Chu-Tan formula, has been used in NSCLC treatment in South China for years. Years of clinical studies have shown that YFSJF improves NSCLC patients' survival and quality of life while also reducing the harmful effects of contemporary therapies. A multicenter, prospective, clinical cohort study with 315 elderly advanced NSCLC patients showed no significant difference in the median overall survival (mOS) and the median time to progression (mTTP) between the Yi-Qi-Chu-Tan group and the chemotherapy group [9]. A case-control study of 62 patients with advanced NSCLC demonstrated that the Yi-Qi-Chu-Tan formula could improve the mOS and the median progression-free survival (mPFS) [10]. In a retrospective study of 376 patients with epidermal growth factor receptor (EGFR) wild-type advanced NSCLC, it was discovered that combining YFSJF with chemotherapy could improve the mOS compared to chemotherapy alone [11]. Another retrospective analysis of 90 patients with EGFR mutant-type advanced NSCLC showed that combining targeted therapy with YFSJF could extend PFS and reduce the side effects of targeted therapy [12]. Other clinical studies reported that combining YFSJF with chemotherapy could reduce fatigue and enhance the quality of life [13–15]. The mechanism of YFSJF for treating NSCLC deserves more attention because of its valid clinical proof.

**Figure 1:**
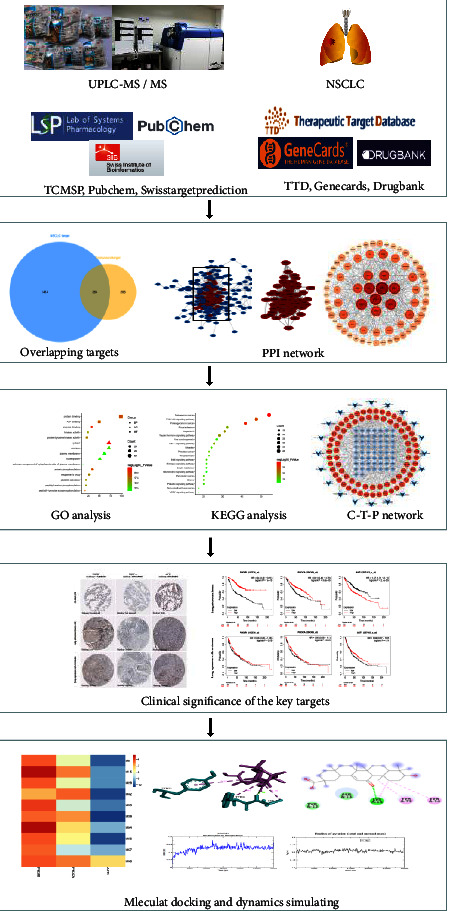
The flowchart of the study.

Scientists are working on multiple aspects of studying TCM, including clinical efficacy, pharmaceutical mechanisms, effective components, extraction separation, and preparation development. The multiple components and targets of TCM make it challenging to figure out its mechanism. With the development of analysis technology, a combination of chromatography and mass spectrometry was invented and applied in various fields, providing the opportunity to separate and analyze complex compositions simultaneously. UPLC-MS/MS has been extensively applied in TCM for chemical analysis, quality control, pharmacokinetics, serum pharmacochemistry, and rapid screening of active components [16–21]. In recent years, network pharmacology has been used to explore the mechanism of TCM, especially for a formula with multiple components [22]. From a holistic standpoint, it can explain the pharmacological impact of a complicated object. This study aimed to use UPLC-MS/MS, network pharmacology, and *in silico* methods to investigate the mechanism of YFSJF against NSCLC.  Figure 1 illustrates the flowchart.

## 2. Materials and Methods

### 2.1. Reagents and Materials

All herbs were purchased from Kangmei pharmacy (Kangmei Pharmaceutical Co., China). HPLC-grade acetonitrile and ethanol were purchased from Fisher Scientific (Pittsburgh, USA), and HPLC-grade formic acid was purchased from Sigma (St. Louis, USA). Water was purified via a purification system (Millipore, Bedford, MA, UAS).

### 2.2. Preparation of YFSJF

The prescribed amount of YFSJF was soaked for 30 min, followed by the addition of 8 and 6 times the volume of water, respectively, for reflux extraction (twice), each for one hour. The filtrate was concentrated to 100 mL, and ethanol was added to the cooled filtrate for a final concentration of 85%. The liquid was filtered, concentrated, and diluted in 100 mL water after 24 hours. The final crude drug concentration was 1.45 g/mL. The sample used in high-performance liquid chromatography-mass spectrometry (HPLC-MS/MS) was a tenfold diluent with ethanol after filtering with a 0.22 *μ*m membrane.

### 2.3. Compound Identification of YFSJF through UPLC-MS/MS

The Shimadzu UPLC XR instrument (Shimadzu Corp, Japan) was employed for chromatographic separation. The following were the chromatographic conditions: chromatographic column, Acclaim 120 C18 (5 *μ*m 4.6 *∗* 250 mm) (Thermo Fisher Scientific, USA); column temperature, 30°C; flow rate, 1 ml/min; mobile phase, acetonitrile (A) and 0.1% aqueous formic acid (v/v) (B); and elution gradient, 5–60% A at 0–60 min, 60–95% at 60–90 min, 95–5% at 90–95 min, and 95–95% at 95–100 min.

A triple TOF-TM 5600 plus hybrid triple quadrupole time-of-flight mass spectrometer (AB SCIEX, USA) equipped with Analyst®TF 1.6 software was utilized for mass spectrometry. The following were the mass spectrometry conditions: electrospray ionization (ESI) source; ion spray voltage, 5500 V in a positive mode and −4500 V in a negative mode; ion source gas 1/2, 55 psi; curtain gas, 35 psi; temperature, 550°C; collision energy, 10 eV; collision energy spread, 20 eV; declustering potential, 80 eV; and scanning range, 50–1500 *m*/*z*. Positive and negative ion modes were used for detection, respectively. The PeakView software equipped with the Chinese medicine mass spectrometry database (AB SCIEX, USA) was used to evaluate the data. By comparing the mass fragments in the database and analyzing the fragmentation pattern, the ingredients of YFSJF were identified.

### 2.4. Screening of Potential Targets for YFSJF in Treating NSCLC

#### 2.4.1. Target Collection of YFSJF

Molecules identified in UPLC-MS/MS were searched in the Traditional Chinese Medicine Systems Pharmacology Database and Analysis Platform (TCMSP, https://tcmspw.com/tcmsp.php). Molecules with anticancer effects or oral bioavailability (OB) less than 20% and drug-likeness (DL) less than 0.15 were enrolled for target screening [23]. As a backup, the PubChem database was used to retrieve the canonical SMILES strings and 3D structures of the specified components (https://pubchem.ncbi.nlm.nih.gov/). The canonical SMILES strings of each selected molecule were imported into the Swiss Target Prediction database to obtain related targets when species was set as *Homo sapiens* (https://www.swisstargetprediction.ch/). The target with a probability of 0.1 or higher was maintained. UniProt was used to correct the target name (https://www.uniprot.org/).

#### 2.4.2. Target Collection of NSCLC

Databases such as the GeneCards (https://www.genecards.org/), Therapeutic Target Database (TTD, https://db.idrblab.net/ttd/), and Drug Bank (https://go.drugbank.com/) were used to search for “non-small-cell lung cancer” and “NSCLC” and get NSCLC targets. The target names were corrected in UniProt.

#### 2.4.3. Potential Target for YFSJF in Treating NSCLC

The overlapping targets of YFSJF and NSCLC were shown in a Venn diagram and were the potential target in treating NSCLC.

### 2.5. Protein-Protein Interaction (PPI) Network Construction

A PPI network without independent nodes was used to elucidate the molecular mechanisms of YFSJF's anti-NSCLC activities by the STRING database (version 10.0, https://www.string-db.org/), with a required confidence level >0.9. The Cytoscape software (version 3.6.1; https://www.cytoscape.org/) was used to examine the network. The key treatment targets were those whose betweenness, closeness, and degree were larger or equivalent to the median, and they were preserved for future visualization. The top 10 degrees in the PPI network were hub targets.

### 2.6. GO and KEGG Pathway Analysis

The Database for Annotation, Visualization, and Integrated Discovery (DAVID, https://david.ncifcrf.gov/) was employed for functional analysis of the key prospective therapeutic targets. The DAVID online tool analyzed target functions using gene ontology (GO) analysis and the Kyoto Encyclopedia of Genes and Genomics (KEGG) pathway analysis.

### 2.7. C-T-P Network Construction

The compounds, main therapeutic targets, and top 20 KEGG pathways were collected in an MS Excel file as a backup. The C-T-P network was created using the Cytoscape software (version 3.6.1; https://www.cytoscape.org/). Hub compounds were among the top ten compounds.

### 2.8. Relationship between Objective Hub Targets and NSCLC Patients

The expression of hub targets in normal and NSCLC tissues was compared using the THPA database (https://www.proteinatlas.org/). The Kaplan–Meier Online website (https://kmplot.com/analysis) was used to investigate the connection between the expression level of hub genes and prognosis in NSCLC. The result was depicted through a survival curve with a hazard ratio (HR).

### 2.9. *In Silico* Verification

#### 2.9.1. Molecular Docking of the Key Targets

Molecular docking technology was used to study the binding interaction between hub compounds and objective hub targets. Compounds and targets were constructed using the PubChem database (https://pubchem.ncbi.nlm.nih.gov/) and the PDB website (https://www.rcsb.org/), respectively. The AutoDock software was used to remove water molecules from the protein structure and hydrogenate it, after which docking pockets were constructed using the protein's ligand. The CB-Dock website (https://cao.labshare.cn/cb-dock/) was used to predict docking pockets for proteins without their ligand. After determining docking pocket parameters, the AutoDock Vina software (1.1.2 version) was used for molecular docking and conformation grading. A 2D combination conformation was generated through Discovery Studio (2019 version).

#### 2.9.2. Molecular Dynamics Simulation of the Key Targets

A molecular dynamics simulation through GROMACS 2021 was used to verify the interaction between hub compounds and objective hub targets. The unconstrained dynamics simulation was run with the CHARMM36 force field and the TIP3P water model. Molecular MOL2 files were converted to str files through the CHARMM General Force Field (CGenFF) website (https://cgenff.umaryland.edu/initguess), and then, str files were converted to topology files through Python 2.7 (script: cgenff_charmm2gmx.py) (https://mackerell.umaryland.edu/charmm_ff.shtml#gromacs). The simulating system employed a dodecahedral solvent box with a 1 ns periodic boundary condition. To balance the system, 500 ps NVT and 1000 ps NPT were used, with a temperature of 300 K and a pressure of 1 bar, followed by a 20∼30 ns simulation. The time increment was 2 fs, and the confirmation was saved every 10 ps.

## 3. Results

### 3.1. Compounds of YFSJF

**Figure 2:**
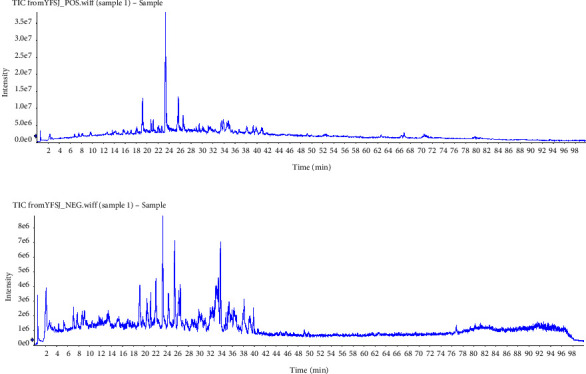
TIC of UPLC-MS/MS for YFSJF. (a) TIC in a positive ion mode. (b) TIC in a negative ion mode.

The complicated ingredients of Chinese decoction make it hard to figure out effective compounds against a specific disease. Compounds were detected using UPLC-MS/MS. The total ion chromatography (TIC) of UPLC-MS/MS is shown in Figure 2. Those with anticancer effects published, OB less than 20%, and DL less than 0.15 were enrolled in target screening. As shown in Table 1, 73 compounds were identified with their herbal attributions indicated by corner markers and 58 compounds (M1-M58) were enrolled for further targeting collection.

### 3.2. Overlapping Targets of YFSJF and NSCLC and the Protein-to-Protein Interaction (PPI) Network

**Figure 3:**
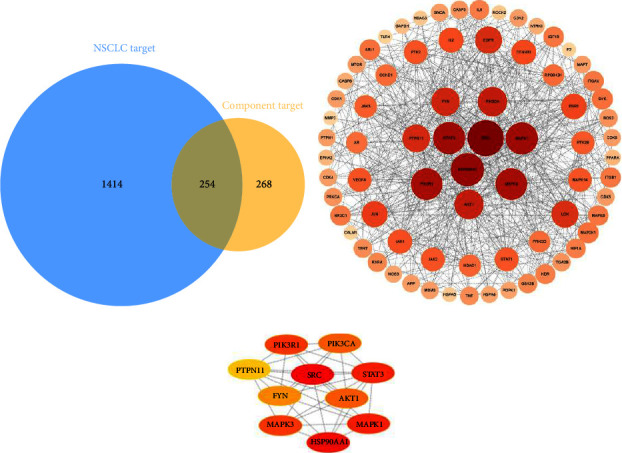
The network analysis of intersection targets of YFSJF and NSCLC. (a) Overlapping targets of YFSJG and NSCLC. (b) PPI network of main overlapping targets. (c) Interrelation of top 10 targets in terms of degrees.

According to the Swiss Target Prediction database, 522 targets were predicted for 58 compounds. NSCLC was estimated to have 1668 targets according to various disease target databases. As shown in Figure 3, 254 targets overlapped and were potential targets for YFSJF to treat NSCLC.

After eliminating independent nodes, the Cytoscape software created a PPI network with 223 nodes and 1288 edges based on 254 overlapping targets. To obtain additional parameters, CytoNCA was used to analyze the network. We screened out 74 main targets whose betweenness, closeness, and degree were greater than or equal to the median (≥123.0941842, ≥0.358642973, ≥8, respectively). Based on key 74 targets, a PPI network with 74 nodes and 640 edges was generated, as shown in Figure 2(b). The degree is determined by the depth of the color or the size of the node. The top 10 in terms of degrees were hub targets, whose interrelation is shown in Figure 2(c). The top 10 targets in terms of degree were PTPN11 (29 degrees), FYN (30 degrees), AKT1 (32 degrees), PIK3CA (32 degrees), PIK3R1 (36 degrees), MAPK3 (36 degrees), STAT3 (37 degrees), MAPK1 (37 degrees), HSP90AA1 (39 degrees), and SRC (44 degrees).

### 3.3. Functional Enrichment Analysis

**Figure 4:**
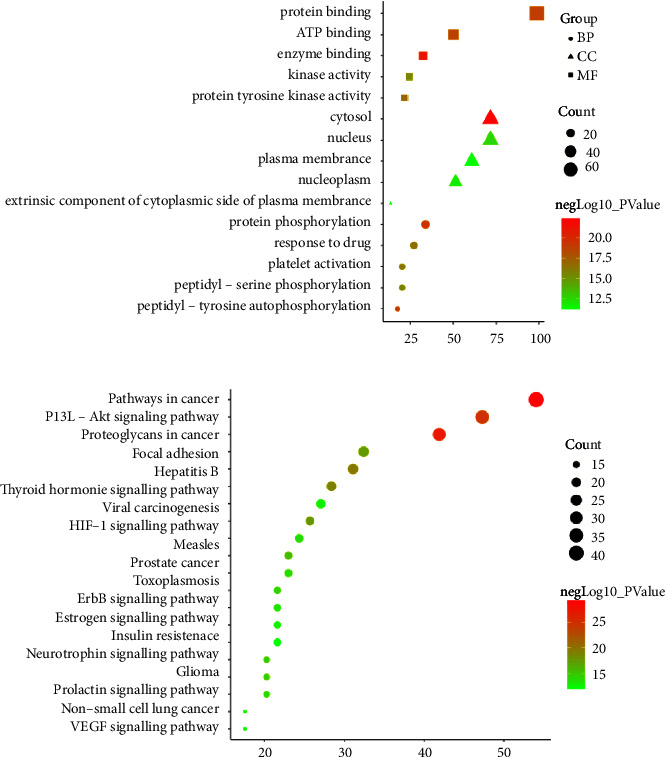
Functional enrichment analysis of potential targets for YFSJF against NSCLC. (a) Top 5 BP/CC/MF with *p* values of 74 targets. (b) Top 20 KEGG pathways with *p* values of 74 targets.

**Figure 5:**
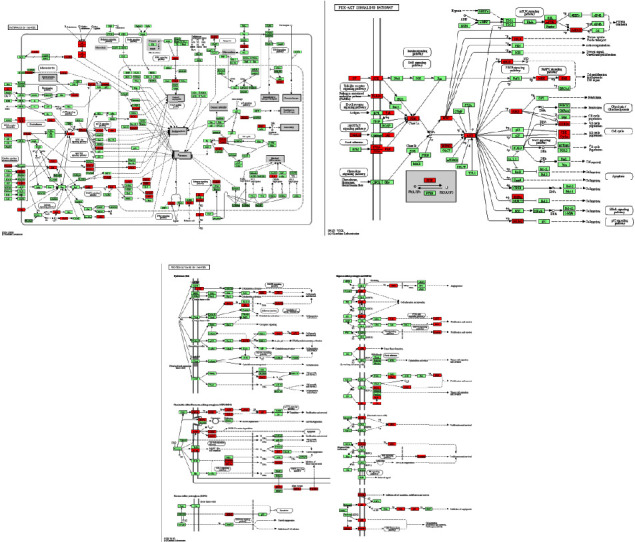
Top 3 pathways in target counts related to YFSJF. (a) Pathways in cancer. (b) PI3K-AKT signaling pathway. (c) Proteoglycans in cancer. Potential targets that are relevant to YFSJF were colored in red.

Gene ontology (GO) and the Kyoto Encyclopedia of Genes and Genomics (KEGG) pathway analysis based on 74 main targets were used to further explore the mechanism of YFJSF for NSCLC.  Figure 4(a) shows the top 5 *p* values in the biological process (BP), cellular component (CC), and molecular function (MF). The major BPs were protein phosphorylation, peptidyl-tyrosine autophosphorylation, response to a drug, platelet activation, and peptidyl-serine phosphorylation. The key CCs were cytosol, nucleus, nucleoplasm, plasma membrane, and the extrinsic components of the plasma membrane. Enzyme binding, protein binding, ATP binding, protein tyrosine kinase activity, and kinase activity were the key MFs. The top 20 *p* values in the KEGG signaling pathway are depicted in Figure 4(b), with details shown in Table 2. Several pathways were related to cancer occurrence and development, including cancer pathways, the PI3K-AKT signaling pathway, and cancer proteoglycans. The table contains more information on the KEGG pathway analysis.  Figure 5 demonstrates the top 3 pathways in target counts related to YFSJF. Because the PI3K-AKT route was found in other pathways, including cancer pathways and cancer proteoglycans (Figure 5), it was assumed to be the primary mechanism. After analyzing the relationship between the top 10 targets and the KEGG pathway results, PIK3R1, PIK3CA, and AKT1, which occupied a critical position in the PIK3-AKT signaling pathway, were considered the three key targets.

### 3.4. Compound-Target-Pathway (C-T-P) Network

**Figure 6:**
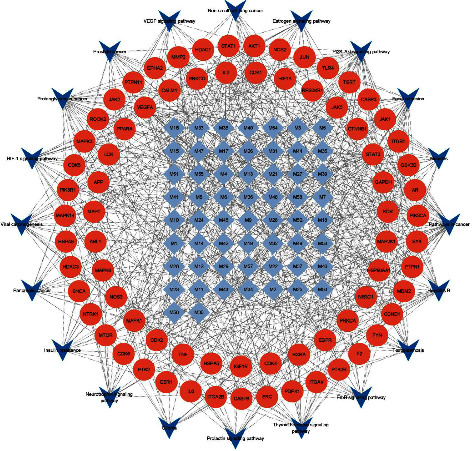
C-T-P network demonstrated the mechanism of YFSJF against NSCLC.

A C-T-P network (Figure 6) was established to demonstrate the mechanism behind YFSJF based on the above data. In YFSJF, the top 10 compounds filtered through cytoHubba were considered hub ingredients: quercetin, fraxin, liquiritigenin, glycyrrhetinic acid, naringenin, liquiritin, imperatorin, peimine, imperialine, and ginsenoside Rb1.

### 3.5. Effects of Key Targets on NSCLC Patients

**Figure 7:**
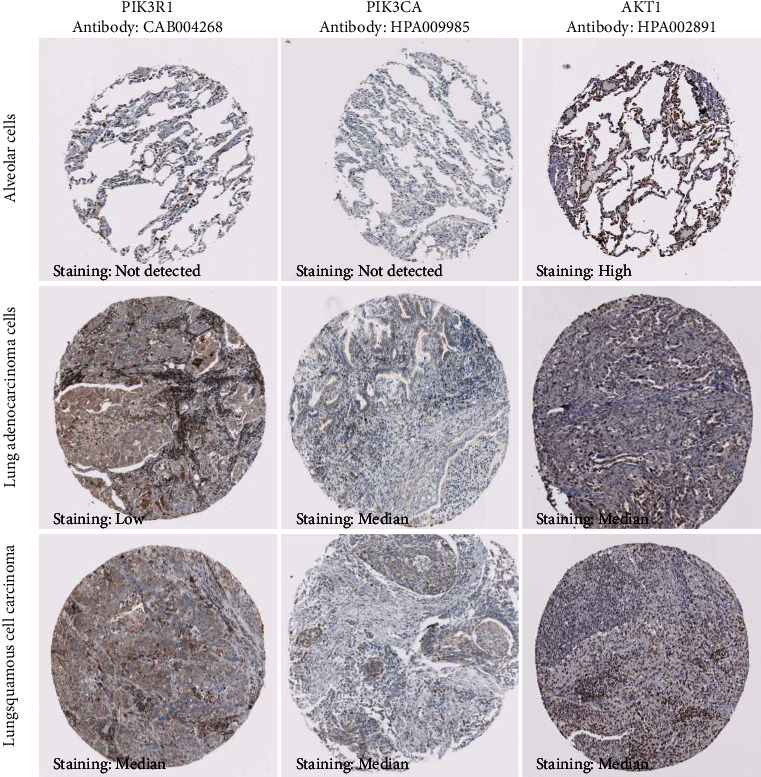
Expression of PIK3R1, PIK3CA, and AKT1 in normal and NSCLC tissue.

The expression of the three major targets in tissue was investigated using the Human Protein Atlas (THPA) database. As shown in Figure 7, the expression levels of the three targets were distinguished between normal and NSCLC tissues. PIK3R1 and PIK3CA were not detected in normal alveolar cells but were found in lung adenocarcinoma and squamous cell carcinoma with lower or medium expression. AKT1 expression was found to be high in normal alveolar cells but average in lung adenocarcinoma and squamous cell carcinoma. Different expressions suggest that PIK3R1, PIK3CA, and AKT1 may play a role in the occurrence and progression of NSCLC.

**Figure 8:**
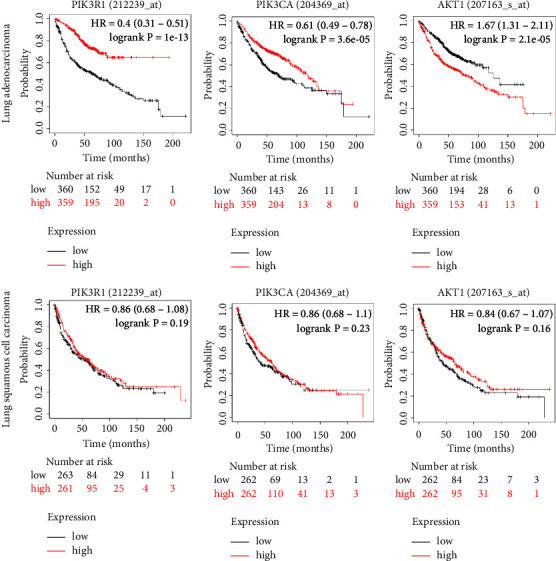
Relationship between 3 key targets and NSCLC prognosis.

The Kaplan–Meier plotter online database was used to analyze the correlation between three key targets and the prognosis of NSCLC (Figure 8). The survival curve indicated that the expression of PIK3R1, PIK3CA, and AKT1 correlated with the mOS of lung adenocarcinoma (*p* < 0.05). The mOS of the group with high PIK3R1 and PIK3CA expression was better than that of the group with low PIK3R1 and PIK3CA (HR = 0.4, HR = 0.61, respectively). The mOS result was reversed for different expressions of AKT1 (HR = 1.67). In the case of lung squamous cell carcinoma, the curves for different expressions in 3 key targets appeared to separate, particularly for AKT1. The separating tendency indicated a correlation between the target and the mOS in lung squamous cell carcinoma, but there was no statistical significance.

### 3.6. Molecular Docking and Molecular Dynamics Simulation

**Figure 9:**
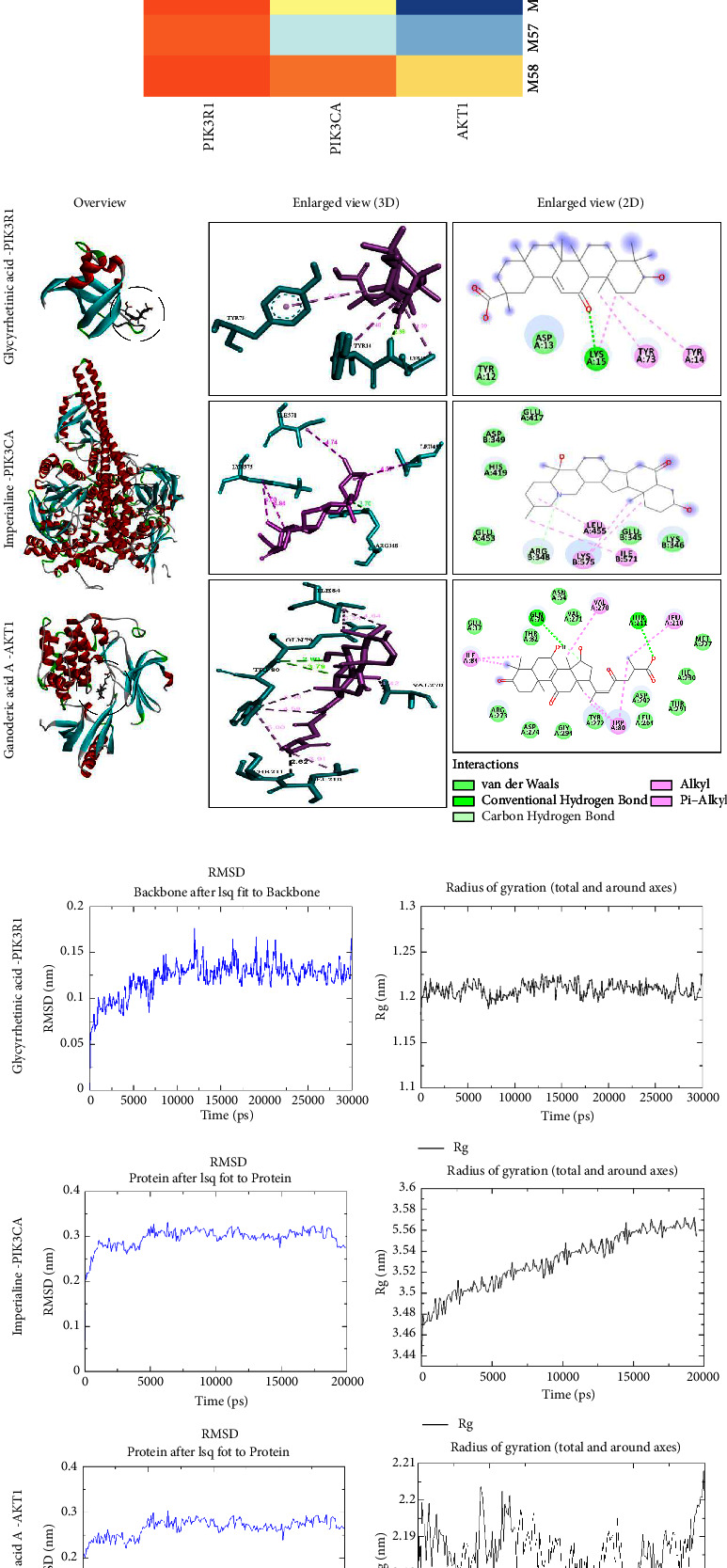
(a) The heatmap of binding affinity between 10 hub compounds and three key targets (PDB ID: PIK3R1-3I5R, PIK3CA-4JPS, and AKT1-6HHH). (b) The detailed molecular docking result of the strongest binding affinity pair. (c) The molecular dynamics simulation results of protein RMSD and Rg.

The binding affinity of ten hub compounds and three major targets was investigated using molecular docking. Generally, binding affinities of less than −5 kcal/mol are considered, indicating a good confirmation intersection. The result was visualized through a heatmap (Figure 9(a)), in which a deeper blue color indicated a stronger binding affinity. All 10 hub compounds were significant for PIK3R1, PIK3CA, and AKT1 docking, with M32 (glycyrrhetinic acid), M57 (imperialine), and M39 (ganoderic acid A) possessing the strongest binding affinity, respectively.  Figure 9(b) shows the structure of the most potent binding molecules with three important targets. It suggests that the molecule may combine with protein through a hydrogen bond, carbon-hydrogen bond, alkyl, and pi-alkyl. The molecular dynamics simulation showed that the three systems became stable between 20∼30 ns and that all proteins' root mean square error (RMSD) fluctuated around 0.1 nm. The radius of gyration (Rg) gradually increased more or less as the simulation progressed, indicating that the molecule may hinder protein by uncoiling it.

## 4. Discussion

TCM's abundance of herbs and diverse formulas create a blue ocean for medical study, attracting an increasing number of researchers. This study discovered multiple ingredients, targets, and signal pathways for YFSJF against NSCLC.

The discovery of 73 YFSJF components using UPLC-MS/MS established the groundwork for additional research. Fifty-eight filtered compounds were enrolled in network pharmacology analysis. The PPI network was built using the intersection of compound targets and NSCLC. PTPN11, FYN, AKT1, PIK3CA, PIK3R1, MAPK3, STAT3, MAPK1, HSP90AA1, and Src were promising targets for YFSJF against NSCLC by the PPI. They are mainly involved in PI3K-AKT signaling and MAPK signaling. Src and Fyn are members of the Src family kinases of nonreceptor tyrosine kinases, mediating different intracellular signaling pathways. Src family kinases have been identified as important in terms of progression, invasion, metastasis, bone pain, and drug resistance in human cancer, including NSCLC [24–26]. Src and Fyn are activators of PI3K and can mediate the PI3K/AKT/mTOR pathway [27]. PIK3R1, PIK3CA, and AKT1 play a critical role in the PI3K/AKT pathway. Type I PI3K is a heterodimer, consisting of PIK3R1 and PIK3CA, closely associated with cancer. PI3K activation can phosphorylate PIP2 into PIP3, then, PIP3 recruits PDK1 and AKT1 to the plasma membrane, and PIP3 activates AKT1. The activation of AKT1 further stimulated downstream pathways to promote cancer cell proliferation, invasion, and metastasis angiogenesis [28, 29]. MAPK1 and MAPK3 (also known as ERK2 and ERK1, respectively) are serine-threonine protein kinases, which are found downstream of classical MAPK signaling, and can promote cell proliferation, cell cycle, and adhesion [30]. Besides, there is cross talk between the two signaling pathways. Inhibition of the PI3K/AKT signaling pathway leads to the compensatory generation of the MAPK/ERK signaling pathway and vice versa [31, 32]. PI3K inhibitors inhibit AKT and activate B-Raf (upstream of the MAPK pathway), boosting MAPK pathway activation [33]. By inhibiting mTOR and P70S6K (downstream of the PI3K-AKT pathway), mTOR inhibitors can activate the MAPK pathway via a feedback loop [34]. In NSCLC patients, aberrant activation of PI3K/AKT and MAPK pathways regulates tumor occurrence, development, and treatment resistance. Hence, multiple inhibitors of two pathways are being developed and clinically tested for NSCLC therapy [35, 36]. For instance, HH2710, an ERK1/2 inhibitor, was approved by the FDA in September 2019 to treat cancer, including NSCLC with a gene mutant of the MAPK pathway, and subsequently approved by the CDE in March 2020.

The gene ontology biological process (GO-BP) analysis showed that YFSJF is primarily involved in protein/amino acid phosphorylation and autophosphorylation. Protein phosphorylation is one of the most prevalent post-translational modifications. It regulates protein function and various pathways. Enzymes catalyzing protein phosphorylation are called protein kinases. Protein kinases are the principal hub targets of YFSJF, including Src, Fyn, PI3K, AKT, and ERK1/2. They can phosphorylate downstream signals to activate appropriate pathways [37]. The GO-CC analysis result showed that YFSJF is mainly related to intracellular constituents, such as cytosol, nucleus, and nucleoplasm. It was consistent with hub target-related pathways, as PI3K/AKT and MAPK pathways are intracellular [37]. The GO-MF analysis result showed that hub targets were uniform. The KEGG analysis result revealed that YFSJF produced therapeutic effects on NSCLC by regulating multiple pathways (e.g., pathways in cancer, PI3K-AKT signaling pathway, proteoglycans in cancer, HIF-1 signaling pathway, ErbB signaling pathway, and VEGF signaling pathway). They were closely related to the tumor microenvironment (e.g., extracellular matrix stability and hypoxia) and tumor behavior (e.g., invasion and metastasis), and the PI3K-AKT pathway was involved in other pathways [38–45].

Ten YFSJF hub chemicals were identified using the C-T-P network. Combining PPI and KEGG pathway results, we infer that YFSJF may mainly treat NSCLC by targeting PIK3R1, PIK3CA, and AKT1 and regulating the PI3K/AKT pathway. As a result, prognostic analysis and molecular docking were applied to the three primary targets. The results showed that PIK3R1, PIK3CA, and AKT1 expressions could distinguish between normal and NSCLC tissues and might be used as prognostic markers in NSCLC. Overexpression of PIK3R1 was shown to be significantly associated with a poor prognosis in adenocarcinoma [46]. Studies have shown that PIK3CA mutations may confer a survival advantage [47, 48]. Other studies have found that PIK3CA gene mutations are associated with poor OS and reduced PFS of EGFR-TKI treatment in different subtypes of NSCLC [49–52]. Overexpression of p-AKT may help cancer patients live longer [53]. Finally, molecular docking and dynamics simulations validated the hypothesis that YFSJF inhibits PIK3R1, PIK3CA, and AKT1 to suppress the PI3K/AKT pathway and fight NSCLC. After further literature review, it was found that the antitumor role of hub chemicals has been reported by many researchers, especially for lung cancer [54–59]. For instance, ginsenoside compound K, the major intestinal bacterial metabolite of ginsenoside Rb1, can induce apoptosis and autophagy in NSCLC cells by regulating the AMPK/mTOR pathway [54]. Naringenin can induce Bax-mediated mitochondrial apoptosis in human lung adenocarcinoma A549 cells [55]. Liquiritigenin can suppress lung adenocarcinoma A549 cell migration via the PI3K/AKT pathway [56]. 18*β*-Glycyrrhetinic acid can induce apoptosis and G2/M cell cycle arrest and inhibit migration via ROS/MAPK/STAT3/NF-*κ*B signaling pathways in A549 lung cancer cells [57].

## 5. Conclusion

The study verified the characteristics of multiple ingredients, targets, and pathways for YFSJF against NSCLC, with PIK3R1, PIK3CA, and AKT1 identified as the major targets associated with NSCLC patients' prognosis. The PI3K/AKT pathway is identified as the key pathway. Hub compounds demonstrated a good affinity for the three key targets. To combat NSCLC, YFSJF may inhibit PIK3R1, PIK3CA, and AKT1 to suppress the PI3K/AKT pathway. This study provides a solid foundation for future YFSJF mechanism research.

## Figures and Tables

**Table 1:** Identification of chemical constituents in YFSJ decoction.

No./mol ID	Found at RT (min)	Formula	Selected mode	Name	Error (ppm)	Found at mass (Da)	Extraction mass (Da)
1^f^	2.04	C_4_H_6_O_5_	[M − H]^−^	Malic acid	0.6	133.01434	133.01425
2^b,f^	2.08	C_6_H_8_O_7_	[M − H]^−^	Citric acid	0.4	191.01981	191.01973
3^a^	2.08	C_7_H_12_O_6_	[M − H]^−^	Quinic acid	0	191.0561	191.05611
4^a,c,d,f^	2.1	C_4_H_6_O_4_	[M − H]^−^	Amber acid	1.7	117.01953	117.01933
5^c,f^	2.1	C_5_H_9_NO_4_	[M − H]^−^	Glutamic acid	1.5	146.04611	146.04589
6^a^	2.15	C_7_H_10_O_5_	[M − H]^−^	Shikimic acid	−2.4	173.04514	173.04555
7/M1^a^	2.92	C_16_H_18_O_9_	[M − H]^−^	Neochlorogenic acid	−0.9	353.08748	353.08781
8/M2^a^	2.92	C_16_H_18_O_9_	[M − H]^−^	Cryptochlorogenic acid	−0.9	353.08748	353.08781
9/M3^a^	2.92	C_16_H_18_O_9_	[M − H]^−^	Chlorogenic acid	−1	353.08748	353.08782
10^e,f^	3.87	C_5_H_4_N_4_O	[M + H]^+^	6-Hydroxypurine	−6.6	137.04489	137.04579
11^e,f^	5.01	C_5_H_5_N_5_	[M + H]^+^	Adenine	−5.1	136.06107	136.06177
12^e,f^	5.2	C_6_H_9_N_3_O_2_	[M + H]^+^	Histidine	−5.5	156.07589	156.07675
13/M4^e^	7.21	C_10_H_13_N_5_O_3_	[M + H]^+^	Cordycepin	−4.3	252.10804	252.10911
14/M5^a,c,e,f^	7.72	C_9_H_8_O_4_	[M − H]^−^	Caffeic acid	0.3	179.03503	179.03498
15^c^	7.85	C_6_H_6_O_3_	[M + H]^+^	5-Hydroxymethylfurfural	−6.6	127.03813	127.03897
16^f,g^	8.51	C_6_H_14_N_4_O_2_	[M + H]^+^	L(+)-arginine	−4.4	175.11819	175.11896
17^f^	8.73	C_11_H_12_N_2_O_2_	[M − H]^−^	L-tryptophan	0.7	203.08275	203.0826
18/M6^d^	9.38	C_10_H_10_O_4_	[M − H]^−^	Isoferulic acid	2.6	193.05113	193.05063
19/M7^d,e,f^	9.38	C_10_H_10_O_4_	[M − H]^−^	Ferulic acid	2.6	193.05113	193.05063
20/M8^f^	9.44	C_15_H_10_O_7_	[M − H]^−^	Quercetin	−3.5	301.03434	301.03539
21/M9^d^	10.49	C_2_8H_34_O_15_	[M − H]^−^	Hesperidin	−3.6	609.18028	609.18248
22/M10^b^	10.5	C_21_H_18_O_12_	[M − H]^−^	Luteolin-7-O-*β*-D-glucuronide	−3	461.07119	461.07256
23/M11^a^	10.7	C_25_H_24_O_12_	[M − H]^−^	Isochlorogenic acid B	−2.3	515.11833	515.1195
24/M12^a^	10.7	C_25_H_24_O_12_	[M − H]^−^	Isochlorogenic acid C	−2.3	515.11833	515.1195
25/M13^a^	10.7	C_25_H_24_O_12_	[M − H]^−^	Isochlorogenic acid A	−2.3	515.11833	515.1195
26/M14^d^	10.74	C_22_H_22_O_9_·HCOOH	[M − H]^−^	Ononin +HCOOH	−2.3	475.12351	475.12459
27^f^	11.54	C_7_H_6_O_3_	[M − H]^−^	Protocatechuic aldehyde	2.5	137.02476	137.02442
28/M15^a^	12.47	C_16_H_18_O_10_	[M − H]^−^	Fraxin	−1	369.08234	369.08272
29/M16^a^	13.28	C_10_H_8_O_5_	[M + H]^+^	Fraxetin	−4.1	209.04359	209.04445
30/M17^a^	13.44	C_9_H_6_O_4_	[M − H]^−^	Esculetin	1.5	177.01959	177.01933
31^g,h^	14.73	C_17_H_20_N_4_O_6_	[M + H]^+^	Vitamin B2	−5.7	377.14341	377.14555
32/M18^f^	15.95	C_26_H_28_O_14_	[M − H]^−^	Isoschaftoside	−2.8	563.13904	563.14063
33/M19^f^	15.95	C_26_H_28_O_14_	[M − H]^−^	Schaftoside	−2.8	563.13904	563.14063
34/M20^d^	16.17	C_13_H_18_O_7_	[M − H]^−^	Gastrodin	−1	285.0977	285.09798
35/M21^e^	16.57	C_9_H_8_O_2_	[M − H]^−^	Cinnamic acid	0.1	147.04517	147.04515
36/M22^d,e^	18.21	C_8_H_8_O_3_	[M − H]^−^	Vanillin	4.7	151.04078	151.04007
37/M23^a,b^	18.23	C_27_H_30_O_16_	[M − H]^−^	Rutin	−1.8	609.14498	609.1461
38/M24^a^	19.04	C_21_H_22_O_11_	[M − H]^−^	Astilbin	−1.6	449.10821	449.10893
39/M25^f^	19.18	C_21_H_22_O_9_	[M − H]^−^	Liquiritin	−2.4	417.1181	417.1191
40/M26^f^	19.18	C_21_H_22_O_9_	[M − H]^−^	Isoliquiritin	−2.4	417.1181	417.11911
41/M27^a,b,c^	19.26	C_21_H_20_O_12_	[M − H]^−^	Isoquercitrin	−1.8	463.08735	463.0882
42/M28^f^	19.38	C_15_H_12_O_4_	[M + H]^+^	Liquiritigenin	−5.3	257.07948	257.08084
43/M29^a^	19.52	C_21_H_20_O_12_	[M + H]^+^	Hyperin	−2.7	465.10152	465.10275
44/M30^a^	20.47	C_11_H_10_O_5_	[M − H]^−^	Isofraxidin	3	221.04622	221.04555
45/M31^f^	20.5	C_42_H_62_O_16_	[M − H]^−^	Glycyrrhizic acid	−1.3	821.39547	821.39653
46/M32^f^	21.29	C_30_H_46_O_4_	[M − H]^−^	Glycyrrhetinic acid	9	469.33654	469.33232
47^c^	21.61	C_26_H_32_O_11_	[M − H]^−^	Pinoresinol glucoside	−1.3	519.18648	519.18718
48/M33^f^	22.07	C_15_H_12_O_5_	[M + H]^+^	Naringenin	−3.7	273.07474	273.07576
49/M34^a^	22.07	C_27_H_32_O_14_	[M − H]^−^	Naringin	−2.3	579.17062	579.17193
50/M35^h^	24.39	C_48_H_76_O_19_	[M − H]^−^	Ginsenoside Ro	−1.4	955.48948	955.49083
51/M36^h^	25.48	C_48_H_82_O_18_·HCOOH	[M − H]^−^	Ginsenoside Re +HCOOH	−2.4	991.54598	991.54833
52/M37^h^	25.48	C_48_H_82_O_18_·HCOOH	[M − H]^−^	Ginsenoside Rd +HCOOH	−2.4	991.54598	991.54833
53/M38^g^	26.68	C_42_H_66_O_14_	[M − H]^−^	Chikusetsusaponin-IVa	−0.9	793.43727	793.43798
54/M39^g^	33.1	C_30_H_44_O_7_	[M − H]^−^	Ganoderic acid A	−1	515.3009	515.30144
55/M40^g^	33.32	C_32_H_44_O_9_	[M − H]^−^	Ganoderic acid H	−1.1	571.29063	571.29125
56/M41^h^	33.5	C_42_H_72_O_14_	[M − H]^−^	Pseudoginsenoside F11	−1.1	799.48404	799.48491
57/M42^h^	33.51	C_42_H_72_O_14_·HCOOH	[M − H]^−^	Ginsenoside Rf +HCOOH	−1	845.4896	845.49041
58/M43^h^	33.86	C_54_H_92_O_23_·HCOOH	[M − H]^−^	Ginsenoside Rb1 +HCOOH	−2.9	1153.5978	1153.60112
59/M44^h^	34.21	C_36_H_62_O_10_	[M − H]^−^	Pseudoginsenoside-RT5	−1	653.42637	653.42705
60/M45^h^	34.85	C_53_H_90_O_22_	[M − H]^−^	Ginsenoside Rb2	−1.7	1077.58328	1077.58513
61/M46^h^	34.85	C_53_H_90_O_22_	[M − H]^−^	Ginsenoside Rb3	−1.7	1077.58328	1077.5851
62/M47^h^	34.85	C_53_H_90_O_22_	[M − H]^−^	Ginsenoside Rc	−1.7	1077.58328	1077.5851
63/M48^h^	35.43	C_42_H_72_O_13_·HCOOH	[M − H]^−^	Ginsenoside Rg3 +HCOOH	−1	829.49467	829.4955
64/M49^h^	35.44	C_42_H_72_O_13_	[M − H]^−^	Ginsenoside Rg2	0	783.49006	783.49002
65/M50^h^	36.07	C_36_H_62_O_9_·HCOOH	[M − H]^−^	20(R)-Ginsenoside Rh1 +HCOOH	−1.4	683.43664	683.43761
66/M51^h^	36.07	C_36_H_62_O_9_·HCOOH	[M − H]^−^	Ginsenoside F1 +HCOOH	−1.4	683.43664	683.43759
67/M52^a,e^	47.76	C_15_H_20_O_3_	[M − H]^−^	Atractylenolide III	0.6	247.13412	247.13397
68/M53^f^	56.42	C_16_H_14_O_4_	[M + H]^+^	Isoimperatorin	−4.1	271.09538	271.09649
69/M54^f^	56.42	C_16_H_14_O_4_	[M + H]^+^	Imperatorin	−4.1	271.09538	271.09649
70/M55^e^	66.33	C_27_H_41_NO_3_	[M + H]^+^	Peimisine	−7.1	428.31289	428.31593
71/M56^e^	70.61	C_27_H_45_NO_3_	[M + H]^+^	Peimine	−6.5	432.34441	432.34721
72/M57^e^	79.93	C_27_H_43_NO_3_	[M + H]^ + ^	Imperialine	−5.5	430.32917	430.33156
73/M58^e^	80.52	C_18_H_32_O_2_	[M − H]^−^	Linoleic acid	0.9	279.23322	279.23296

*Note.* ^a^Herba Sarcandrae; ^b^Bombyx batryticatus; ^c^Ranunculi Ternati radix; ^d^Pseudobulbus Cremastrae seu Pleiones; ^e^Fritillariae Thunbergii Bulbus; ^f^Rhizoma Pinelliae praeparatum; ^g^Ganoderma; ^h^Panacis Quinquefolii radix.

**Table 2:** KEGG analysis of potential target genes of YFSJF for NSCLC.

Serial number	Term	Count	%	*p* value
hsa05200	Pathways in cancer	40	54.05	6.33*E − *30
hsa04151	PI3K-AKT signaling pathway	35	47.30	1.57*E − *25
hsa05205	Proteoglycans in cancer	31	41.89	8.18*E − *28
hsa04510	Focal adhesion	24	32.43	3.83*E − *18
hsa05161	Hepatitis B	23	31.08	2.61*E − *20
hsa04919	Thyroid hormone signaling pathway	21	28.38	1.06*E − *19
hsa05203	Viral carcinogenesis	20	27.03	1.72*E − *13
hsa04066	HIF-1 signaling pathway	19	25.68	2.20*E − *18
hsa05162	Measles	18	24.32	1.85*E − *14
hsa05215	Prostate cancer	17	22.97	3.53*E − *16
hsa05145	Toxoplasmosis	17	22.97	1.48*E − *14
hsa04012	ErbB signaling pathway	16	21.62	7.86*E − *15
hsa04915	Estrogen signaling pathway	16	21.62	5.82*E − *14
hsa04931	Insulin resistance	16	21.62	2.20*E − *13
hsa04722	Neurotrophin signaling pathway	16	21.62	1.08*E − *12
hsa05212	Pancreatic cancer	15	20.27	2.52*E − *15
hsa05214	Glioma	15	20.27	2.52*E − *15
hsa04917	Prolactin signaling pathway	15	20.27	9.36*E − *15
hsa05223	Non-small-cell lung cancer	13	17.57	3.08*E − *13
hsa04370	VEGF signaling pathway	13	17.57	9.21*E − *13

## Data Availability

All the results used to support this work are included in the article. More details were deposited in the Science Data Bank (DOI: 10.11922/sciencedb.01534).
